# Functional Outcome of Open Latarjet Procedure in Non-Athletic Middle-Aged Patients

**DOI:** 10.5704/MOJ.2107.022

**Published:** 2021-07

**Authors:** S Joshi, VKV Rao, UC Shetty, S Rai, S Arora, SR Kumar

**Affiliations:** 1 Department of Orthopaedics, Government Medical College Kota, Kota, India; 2 Department of Orthopaedics, Kundapur Hospital, Kundapur, India; 3 Department of Orthopaedics, Orthocity Hospital, Varanasi, India

**Keywords:** recurrent anterior shoulder dislocation, bony bankart, hill sachs, open latarjet, coracoid graft

## Abstract

**Introduction:**

The movement and steadiness of the shoulder joint is due to both the dynamic and static stabilisers. Recurrent anterior shoulder instability is common due to the Bankart lesion or the Hill Sachs lesion. The bone loss and soft tissue failure due to these lesions causing instability is well compensated by Latarjet procedure which acts by triple blocking effect of the bone graft, the sling effect of the conjoint tendon of subscapularis and the ligament of the coracoacromial ligament stump.

**Materials and methods:**

Middle-aged patients with recurrent anterior shoulder dislocation and a mid-range instability on clinical assessment with an isolated glenoid bone loss of 20% or Bankart lesion with engaging Hill Sachs lesion were selected for the study. The surgical procedure included a subscapularis split to expose the glenoid. The coracoid graft harvested was prefixed with Kirschner wires and placed flush over the glenoid ensuring no medial or lateral overhang and fixed with 4.0mm cancellous screws with the washer. The functional outcome was measured with the ROWE score and ASES score and the movements were evaluated.

**Results:**

A total of 24 patients fulfilled the inclusion criteria. Post-operatively at final follow-up, the mean ROWE score was 97.08 ±8.45 and the mean ASES score was 94.4±9.10. One patient had screw breakage as a complication and another had restriction of movement which was managed with physiotherapy.

**Conclusion:**

Open Latarjet is an effective procedure for recurrent anterior shoulder instability in non-athletic middle-aged patients as a excellent functional outcome was achieved with this technique. We therefore recommend open Latarjet as an alternative to arthroscopic treatment in developing countries where patient affordability and the availability of the resources are the issues.

## Introduction

Shoulder joint is a complex anatomical and biomechanical structure where the smooth functioning of the joint is due to various stabilisers which work in a coordinated and special manner in various stages of motion. Stability of the shoulder is due to static as well as dynamic stabilisers such as glenohumeral articulation, glenohumeral ligaments, labrum, rotator cuff, and deltoid muscle. The bony contact of the glenoid with the head of humerus is only 30% suggesting that the joint has a shallow osseous confinement. So, the major stability of the joint is because of the various soft tissue structures rather than the bony contact^1^. This comes with the advantage of providing a broad range of motion but also predisposes the joint to be dislocated or subluxated due to trauma. The anterior glenoid labrum deepens the glenoid cavity up to 50%, playing a key role in the anteroposterior stability^2^. Therefore, recurrent anterior instability is caused by injuries resulting in the detachment of the labrum from its attachment to glenoid. The anteroinferior detachment of the glenoid labrum described as a Bankart lesion, was established in up to 87% to 100% shoulder dislocations occurring for the first time^3^.

Instability of the shoulder is most often witnessed in the cohort of late teenage to mid-thirties^4^. The high risk of recurrence among young patients is a major concern post primary traumatic anterior shoulder dislocation^5^. The factors increasing the risk of recurrence are glenoid bone loss,^6^ engaging Hill-Sachs lesion, hyperlaxity,^7^ glenoid concavity deficiency,^8^ and a population population below 20 years at the time of first dislocation^7^.

A French surgeon Dr Michel Latarjet in 1954 described the Latarjet procedure for the first time for the treatment of recurrent anterior shoulder dislocation. He described a coracoid process transfer procedure in which, to treat the anterior glenohumeral instability, coracoid’s inferior surface was made to pass through the subscapularis tendon and further secured firmly to the glenoid at its anteroinferior part^9^.

In 1980, Patte *et al* described a modification in the Latarjet procedure that renders stability through the ‘‘triple blocking effect’’, which includes the graft’s osseous effect, the sling effect of the conjoint tendon on the subscapularis, and the ligament effect as far as the coracoacromial ligament stump is concerned^10^.

However, post-operatively after the Latarjet procedure, the prevalence of glenohumeral arthritis ranges from 49% to 71%^11,12^. The various factors associated with increasing the risk of arthritis in patients having undergone treatment for anterior instability include, the time duration between the age at which first dislocation occurs and the age at which the patient is operated for this condition, frequency of pre-operative dislocations, intra-articular hardware, immoderate tightening of anterior tissue, improper positioning of the graft over the glenoid leading lateral overhang, and protracted follow-up^13^.

The intent of the study aims at finding out the functional outcome of open Latarjet procedure for recurrent anterior shoulder dislocation with mid-range instability and a bony Bankart with or without associated Hill Sachs lesion in non-athletic middle age individual in a developing country like India.

## Materials and Methods

The study undertaken was a prospective one performed at our tertiary care centre after being assessed and approved by the Ethical Review Board (ERB). The duration of study was from June 2018 to May 2020.

The criteria for the inclusion of the patients were a diagnosis indicating a recurrent anterior shoulder dislocation, consent to participate in the study, isolated glenoid bone loss of minimum 20% or bony Bankart lesion with engaging Hill Sachs lesion, patients between the age of 35 to 55 years and mid-range instability on clinical assessment.

The criteria for exclusion were atraumatic shoulder dislocation, multidirectional instability of the shoulder, coexistent musculoskeletal injuries in the ipsilateral shoulder, and a known case of epilepsy or those psychological conditions preventing the patients from following the necessary rehabilitation protocols postoperatively, neurological disorders concerning the ipsilateral shoulder and pre-existing glenohumeral arthritis.

**Fig. 1:**
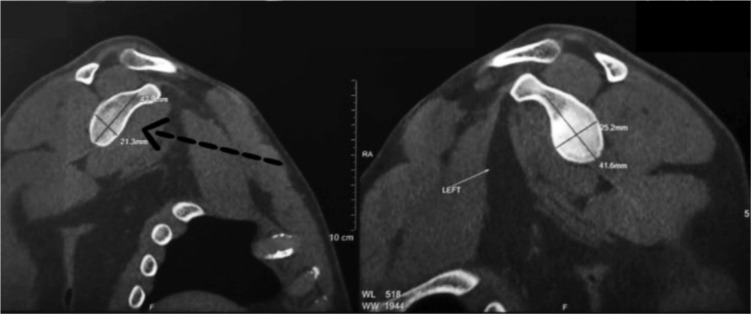
Pre-operative CT showing the glenoid attrition on right side compared to left.

**Fig. 2:**
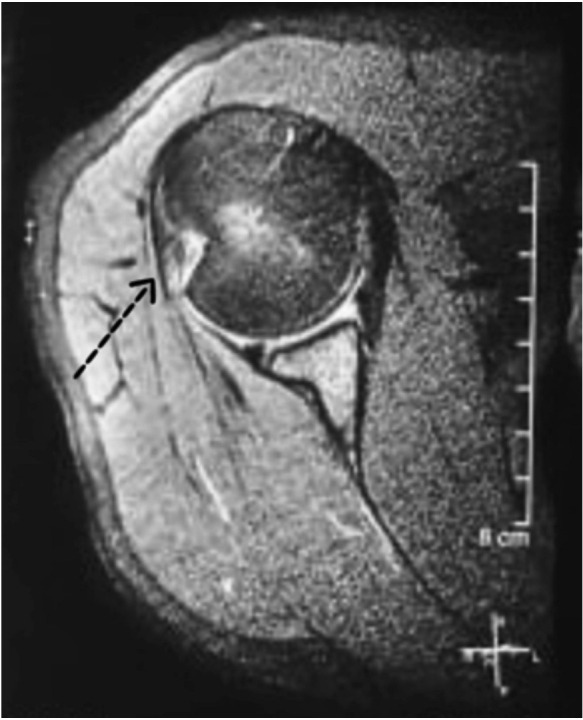
Pre-operative MRI showing Hill Sachs lesion.

Patients’ histories including demographic data, mode of injury, the time-span of instability and the frequency of dislocation episodes pre-operatively were gathered. A detailed physical examination and radiological examination in addition to Rowe score^14^ and ASES^15^ score was comprehensively used for evaluating the patient through an independent surgeon who was not associated with the treatment process. Patients were asked to obtain anteroposterior view and axillary lateral view radiographs pre-operatively and at the time follow-up. Patients pre-operatively were made to undergo a CT scan involving a reconstruction in three dimensions (3D) to bring out the osseous lesion associated with the glenoid and the humeral head (Fig. 1). Pre-operative MRI was obtained in cases where it was feasible (Fig. 2).

In the Rowe’s system, outcomes based on stability, motion and function were graded as excellent, good, fair and poor depending on the scores which ranged from 90-100 points (excellent), 75-89 points (good), 51-74 points (fair), and <50 points (poor).

**Fig. 3:**
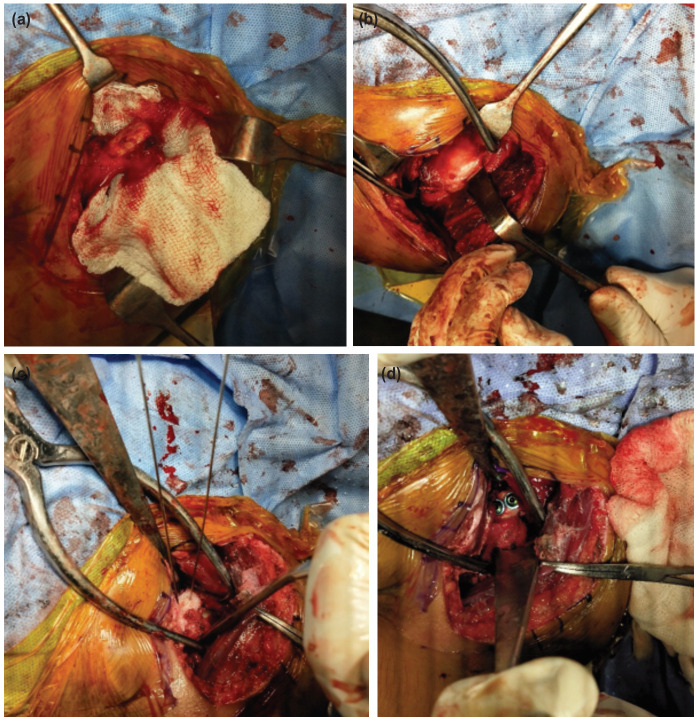
(a) Operative procedure showing Coracoid with attached coracoacromial ligament and conjoint tendon. (b) Operative procedure showing Subscapularis splitting with exposure of glenoid. (c) Operative procedure showing Pre fixed K wires in coracoid graft flushed to glenoid. (d) Operative procedure showing Coracoid graft fixed with 4.0mm cc screw.

The position of the patient was supine on the operating table with a side table for support of the arm. A vertical incision of 7cm to 10cm was put beginning from the tip of the coracoid process which extended up to the anterior border of axilla. The coracoid was exposed after dissecting the deltopectoral interval. This was followed by the blunt dissection of the subcutaneous tissue and incising the clavipectoral fascia aligned to the skin incision. The deltoid musculature is retracted laterally safeguarding the cephalic vein along with it. Throughout the procedure, a meticulous haemostasis is maintained. Laterally, the coracoacromial ligament which is inserted to the coracoid tip was incised 1cm from its insertion (Fig. 3a). Medially, we release the pectoralis minor from its attachment to coracoid. Right at the colligation between the vertical and horizontal aspects (shoulder of coracoid), an osteotomy of the coracoid was done to obtain a graft of 22mm to 25mm from the tip. The inferior cortex of the coracoid graft was removed so that a flat bone surface for concomitant opposition to the surface of the glenoid is created. To access the glenohumeral joint, a subscapularis split approach was used after identifying the three sisters and then dividing the subscapularis horizontally right at the junction of the upper two third and the lower third of the muscle (Fig. 3b). In order to sustain the exposure after splitting the muscle, a subscapularis retractor is consequently used. This exposed the anterior glenohumeral capsule thereby facilitating in performing a vertical capsulotomy at the medial origin after the subscapularis split. To expose the anterior glenoid, a Fukuda retractor was positioned on the head of humerus. With this, the careful excising of the anterior labrum as well as the periosteal sleeve was undertaken. The glenoid’s anteroinferior cortex was freshened thoroughly using a bone file to furnish a flat cancellous bed to boost the graft uptake and healing. Kirschner wires were drilled in the graft at 1cm interval before placing the corocoid on the glenoid. The coracoid graft’s inferior surface was subsequently placed flush with the glenoid’s articular surface and the Kirschner wires were advanced further to fix in the glenoid (Fig. 3c). Two anteroposterior bicortical holes were created with a 2.7mm drill bit at the same site as the Kirschner wires using cannulated drill bit. Confirming the screw length was carried out using a depth gauge and a 4.0mm cannulated cancellous screw with washers was fastened (Fig. 3d) and definitive graft fixation was accomplished. This was followed by confirming the graft position in order to ascertain that it laid flush to the glenoid surface without any medial retracted position or lateral overhang. The arm was externally rotated and the closure of the anterior capsule was done by suturing it to the remaining stump of the coracoacromial ligament on the medial aspect of the graft. The subscapularis was closed by two to three holding sutures.

The shoulder was immobilised in a chest arm immobiliser for 3 weeks after the surgery. Sutures were removed on postoperative day 12. At three weeks post-operatively, passive range of motion physiotherapy was initiated with pendulum exercises. Vulnerable positions were avoided during first six weeks (Overhead throwing movements). Assisted forward flexion up to 90^o^ and assisted abduction up to 90^o^ was started at 1 month and both were was extended beyond 90^o^ after 6 weeks post-operatively. Daily activities were allowed six weeks after the surgery. At three months post-operatively, terminal stretching exercises were allowed.

**Fig. 4:**
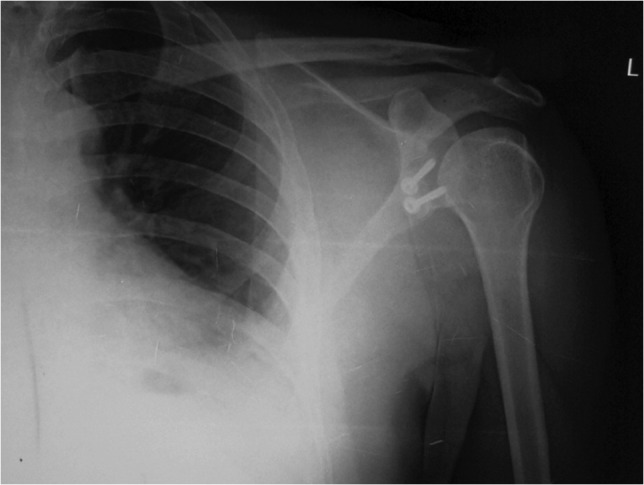
Post-operative radiograph with graft and screws in place.

The patients were analysed in intervals at six weeks, three months, and six monthly up to two years post-operatively. Apprehension test was done three months after surgery. The coracoid graft’s position was examined on the contiguous post-operative films (Fig. 4). A graft with its lateral aspect within 1mm of the glenoid surface was described as flush position.

## Results

A total of 24 patients were considered for the study out of which 19 (76%) were male and 5 (24%) were female. Fourteen patients had recurrent dislocation of the right shoulder and 10 patients had recurrent dislocation of the left shoulder. The mean age was 41.37± 4.18 (range 36 to 49 years). The study population had a mean of 3.66±1.49 episodes of recurrences of dislocations. The mean follow-up was 17.4±2.7 months and ranged from 15 to 24 months. Also, the mean timing of surgery from the first episode of dislocation was 17.79± 4.51 months, and it ranged from 12 months to 24 months. The mean duration of surgery was 68±5.68 minutes. Post-operatively at final follow-up, the mean anterior elevation was 164.16°± 21.04°. The mean abduction was 158.3°±15.75°. The mean external rotation was 54.58°±9.31°. Out of the 24 patients, two had positive apprehension test and the rest 22 patients had negative test at final follow-up. At final follow-up, the mean ROWE score was 97.08±8.45 and mean ASES score was 94.4±9.10 (Table I). Out of 24 patients, 2 (8.33%) patients had post-operative complications. One patient had screw breakage and one had persistent pain and stiffness. Post-operative radiograph showed that all the patients had flush position of the coracoid graft with no medial or lateral overhang.

**Table I:** Showing the functional outcome of open Latarjet procedure at final follow-up

Parameter	Pre-operative Values	Post-operative Values
Anterior elevation (in degree)^a^	152.12+/-20.05	164.16 +/- 21.04
Abduction (in degree)^a^	146.2+/-13.21	158.3 +/- 15.75
External rotation (in degree)^a^	35.14+/-8.25	54.58 +/- 9.31
ROWE score (100)^a^	42.16+/-7.26	97.0 +/- 8.45
ASES score (100)^a^	40.25+/-8.24	94.4+/-9.10
Satisfaction (24)		
Excellent		22 (91.66%)
Good		2 (8.33%)
Fair		0 (0%)
Unsatisfactory		0 (0%)
Apprehension test (24)	24 (100%)	2 (8.33%)

^a^Data are presented as mean +/- SD

The functional outcome in right sided involvement in terms of mean ROWE score was 97.14±8.01 and mean ASES score was 94.42±7.84. On left side, the mean ROWE score was 97±9.48 and mean ASES score was 94.5±11.16. Hence, the functional outcome on both sides were almost similar. The functional outcome in patients of age 40 to 44 years in terms of mean ROWE score was 100 and mean ASES score was 98.12±3.72. The functional outcome in patients of age 45-49 years in terms of mean ROWE score was 99.16±2.04 and mean ASES score was 96.66± 5.16. The functional outcome in patients of age 35-39 years in terms of mean ROWE score was 93.5±12.4 and mean ASES score was 90.2±12.35. There was better outcome in patients between 40 to 45 years but we cannot comment if it’s statistically significant as the sample size is small and needs further study in larger population. The functional outcome in males in terms of mean ROWE score was 96.57±9.43 and mean ASES score was 93.78±10. The functional outcome in females in terms of mean ROWE score was 99±2.23 and mean ASES score was 97±4.47. The females showed better outcome but since we had only five females in the study, it was not possible to comment if they were statistically significant as there was unequal distribution among the two groups.

The functional outcome in patients having isolated Bankart with glenoid bone loss in terms of mean ROWE score was 98.57±2.43 and mean ASES score was 95.71±4.49. The functional outcome in patients with bony Bankart with engaging Hill Sachs lesion in terms of mean ROWE score was 96.47±9.96 and mean ASES score was 93.94±10.56. As only seven patients had isolated Bankart lesions with glenoid bone loss, there is unequal distribution and requires study in a larger population with equal distribution of both groups. However, all the patients were satisfied as far as the procedure was concerned and could reach their pre-injury activity level at their last follow-up.

## Discussion

The shoulder joint is a synovial joint of ball and socket type. It is one of the largest and most complex joint with maximum mobility the body has^16^. Its mobility offers the upper limb with enormous range of motion. But all this comes with a drawback i.e. the instability of the shoulder joint because of the range of movement that it provides. But the rotator cuff muscles, tendons, ligaments, and the glenoid

labrum compensate for this instability. The anterior glenohumeral instability has a prevalence of around 2%^17^. Anterior instability is due to the shoulder’s forced abduction and external rotation causing anterior dislocation^18^. An injury of the anteroinferior glenoid labrum of the shoulder is called as Bankart lesion which causes anterior shoulder dislocation. This leads to a pocket creation right at the glenoid’s anterior aspect which in turn lets the head of humerus to dislocate into it. Hill Sachs lesion is described as a cortical depression found in the humerus head at its posterolateral aspect. Due to anterior dislocation of the shoulder, there is impaction of the head of humerus against the glenoid at its anteroinferior rim. Both these lesions mentioned above lead to recurrent anterior shoulder instability.

In a study by Sugaya *et al*^19^, they observed that 40% of the subjects suffered osseous erosion, and 50% of the subjects had a bony Bankart lesion with recurrent anterior shoulder instability. This suggested that most of the patients with recurrent anterior shoulder instability had an osseous lesion, be it bony Bankart or a Hill Sachs lesion. In cases where the glenoid bone loss is greater than 25%, operative procedure must certainly include a bony reconstruction procedure^20^.

The previous research papers importantly contain the evidence that substantial (above 20% to 25% range)^21-23^ bone loss negatively affects a primary arthroscopic repair. But in a study by Shin *et al*^24^ which was based on a cadaver model stated that, bony restoration procedures should be considered in cases with a bony defect of 15% or more of the widest anteroposterior width of the glenoid because this is reckoned as the critical amount of bone loss where the repair of the soft tissue structures will not be able to restore glenohumeral translation, restrict ROM, and also results in abnormal positioning of the head of humerus for defects parallel to the glenoid’s long axis.

Hence, in our study, we consider a minimum 20% bone loss as a criteria to operate with open Latarjet as arthroscopic procedure does not allow for the preferred bony augmentation necessary to prevent recurrence of subluxation or dislocation.

Latarjet procedure is also used as a bony reconstruction either for the lesion or prophylactically expecting one later because repeated events of anterior shoulder dislocation may engender bony Bankart lesion or Hill-Sachs to grow in size which leads to further instability^25^. Our study showed that the average operating time from incision to closure was around 68 minutes which is around 30 to 40 minutes less compared to arthroscopic Latarjet repair also seen in a comparative study by Zhu *et al*^26^.

Since we did a Latarjet procedure that provided a very good stability due to triple blocking effect, the functional outcome of this study was very good. The mean ROWE score was 97.08 suggesting that most of the patients has an excellent post-operative response to the surgery with a complete range of motion. The mean anterior elevation, abduction and external rotation were 164.16^o^, 158.3^o^, 54.58^o^ which were similar to the functional outcome of open group in a study done by Zhu *et al*^26^. The results were actually comparable to the functional outcome of arthroscopic Latarjet suggesting that open Latarjet provides similar outcome.

**Fig. 5:**
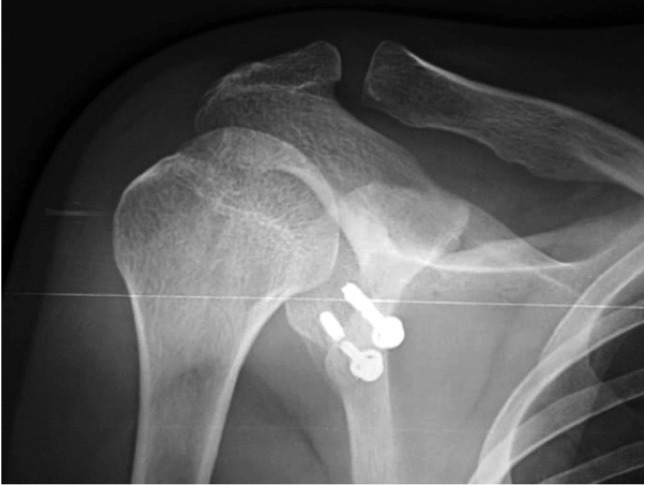
Screw breakage in AP view but with graft in place and showing union.

Our study showed two complications (8.33%), (1) screw breakage which was due to self-fall after having explained to avoid activity without supervision in the 1st 6 weeks (Fig. 5). The patient returned on the 8th week with history of fall on the 42nd post-operative day and on radiography, we found that one of the two screws were broken but the graft was in place, there was union at the graft site and the patient had near complete range of movements. Hence, no intervention was done as the patient was satisfactory with the procedure.

(2) Persistent pain and stiffness as the patient kept the shoulder immobilised for four months without starting any physiotherapy. This was managed by mobilising the shoulder under GA and immediately starting aggressive physiotherapy. The patient later showed improvement in the range of motion with a ROWE score of 70. None of our patients showed any recurrence in the follow-up suggesting that the Latarjet procedure provides excellent stability, in fact comparable to arthroscopic repair as also seen in a study by Zhu *et al*^26^.

The screws were not removed in any case as the patients had a good functional outcome and did not have any complaints and the grafts had showed good union.

Bhatia *et al*^27^ concluded that the medial positioning of the graft was a significant factor for recurrence of dislocation which we avoided by intra-operatively confirming the flush position of the graft against the glenoid. Lateral positioning of the graft will cause post-operative arthritis in a long term as concluded by Allain *et al*^11^. Hence, proper positioning of the graft can prevent recurrence and long term post-operative arthritis. The post-operative radiographs in our study also showed the flush position of the graft in 100% of the cases without any evidence of arthritis at the last follow-up. Since ours is a short term study, it is not possible to comment much on the dislocation arthropathy.

Another study by Ernstbrunner *et al*^28^ also showed that the long term results of arthroscopic Bankart repair had higher rates of reoperation with a second procedure by open Latarjet procedure due to the recurrence of instability. But the open Latarjet group in the study had reoperation not for recurrence of instability but due to persistent pain. It is a known phenomenon which ensues persistent pain after the Latarjet procedure. Only one of our patients had persistence of pain which later reduced after appropriate mobilisation and physiotherapy.

The arthroscopic Latarjet is comparably a perplexing procedure requiring a steep learning curve in addition to a determined degree of technical skill as well as expertise as said by Boileau *et al*^29^ and in a setup of a developing country like India with limited resources, it becomes furthermore difficult. Since our study included non-athletic individuals, the return to sports as evaluated by other studies was not done.

In a meta-analysis by Huley *et al*^30^, comparing open and arthroscopic Latarjet procedure for anterior shoulder instability, they showed no statistically significant results between the two groups as far as ROWE score, the External Range of Motion (EROM), total recurrence, revisions due to recurrence, screw complications, wound infections, graft complications and neurological complications were concerned. But, the persistence of apprehension which was significantly more in arthroscopically operated patients.

A study by Randelli *et al*^31^ comparing the cost analysis and clinical results of open and arthroscopic Latarjet showed excellent and comparable functional outcome results in both groups but they also said that the cost-benefit analysis of the arthroscopic procedure is not very comfortably justified at present. Wedge profile plate currently in use for fixation of coracoid on glenoid and specific instrument set for coracoid graft harvesting are very expensive and cannot be afforded by everyone in a country like India. Arthroscopic repair is out of reach for the general population of India which mainly consists people of middle class and lower class as it is very expensive. We have to serve a population of 1.35 billion with limited resources available. Hence, this study focuses on finding the best alternative for developing nations to achieve similar result.

The limitations of our study were the small sample size, lack of control group of arthroscopically operated patients for anterior shoulder instability to compare our results and short follow-up period. Further studies are needed to address these limitations and to verify our results.

## Conclusion

Considering all the above factors, open Latarjet is an effective procedure for recurrent anterior shoulder instability in non-athletic middle-aged patients as excellent functional outcome were achieved with this technique. We therefore recommend open Latarjet as an alternative to arthroscopic treatment in developing countries where patient affordability and the availability of the resources are the issues.
